# Supplementary material for: PrimPol is required for the maintenance of efficient nuclear and mitochondrial DNA replication in human cells
Source: Nucleic Acids Res. 2019 Jan 31;47(8):4026–38. doi: 10.1093/nar/gkz056 (PMC6486543; doi:10.1093/nar/gkz056)
Supplement: Supplementary Data [file gkz056_supplemental_files.pdf]

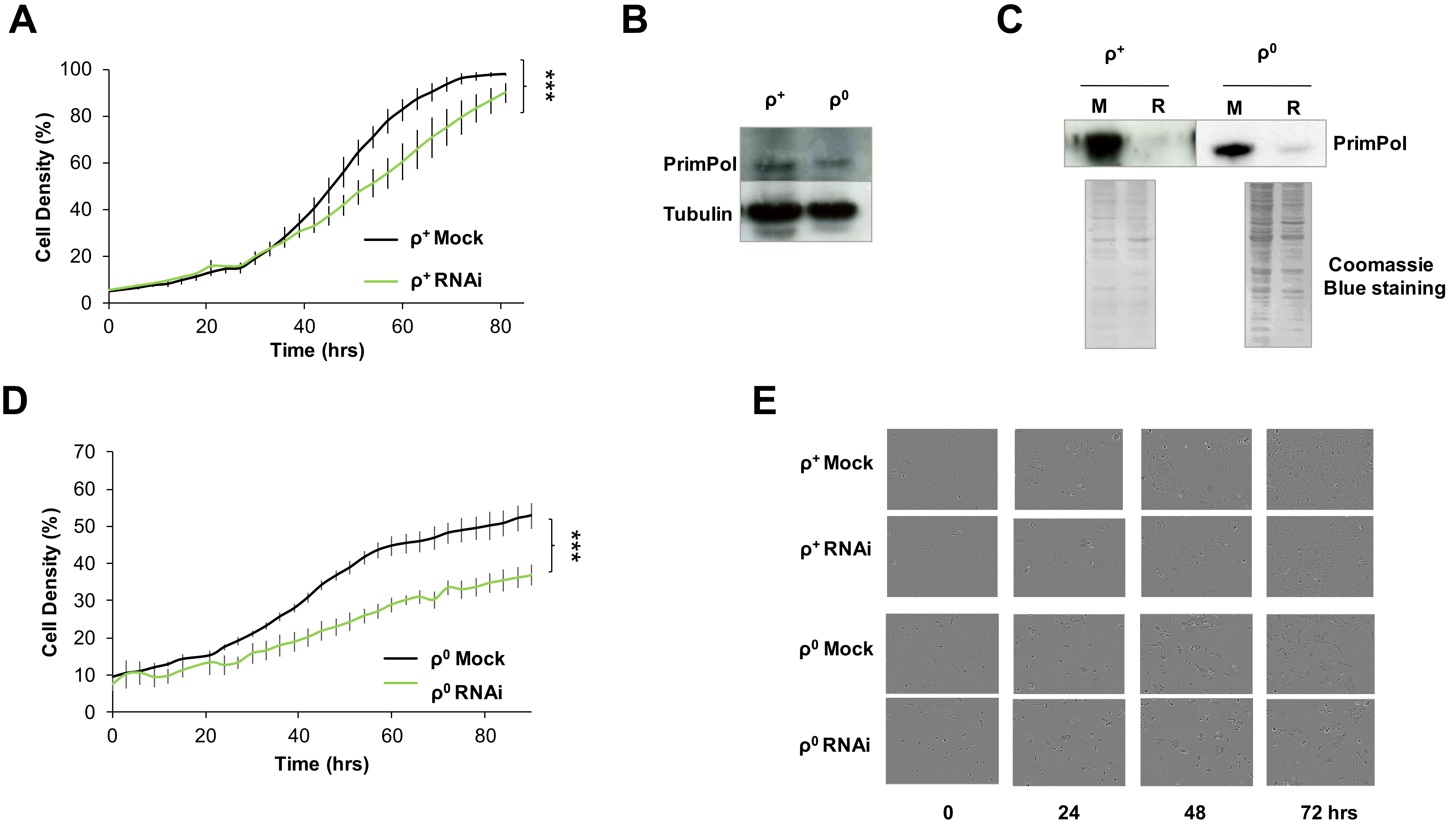

Supplementary Figure 1

Supplementary Figure 1. *PrimPol* RNAi causes a decrease in cell proliferation in both WT and ρ^0 cells. An example growth curve for 143B cells following transfection with siRNA targeted at *PrimPol* or a scrambled control. Images were taken using an IncuCyte™ phase contrast microscope and chart represents an average of two or three individual cell samples grown concurrently with 9 regions analysed per sample at 3 hourly time points error bars show standard deviation across different samples (**A**). PrimPol levels were analysed by western blot of whole cell lysate in wild type (ρ^+) human osteosarcoma cells and those lacking mtDNA (ρ^0), quantified in relation to tubulin levels (n=3) (**B**). PrimPol depletion was confirmed 72 hrs after RNAi treatment (R), compared with mock treated cells (M) by western blot of whole cell lysate with comassie to show similar levels of loading (**C**). Example growth curves for ρ^0 143B cells after *PrimPol* RNAi treatment at time 0 taken using an IncuCyte™ phase contrast microscope (**D**). Each growth curve represents an average of two or three individual cell samples grown concurrently with 9 regions analysed per sample at 3 hourly timepoints. (**E**) shows representative images taken from the microscope at the specific timepoints.

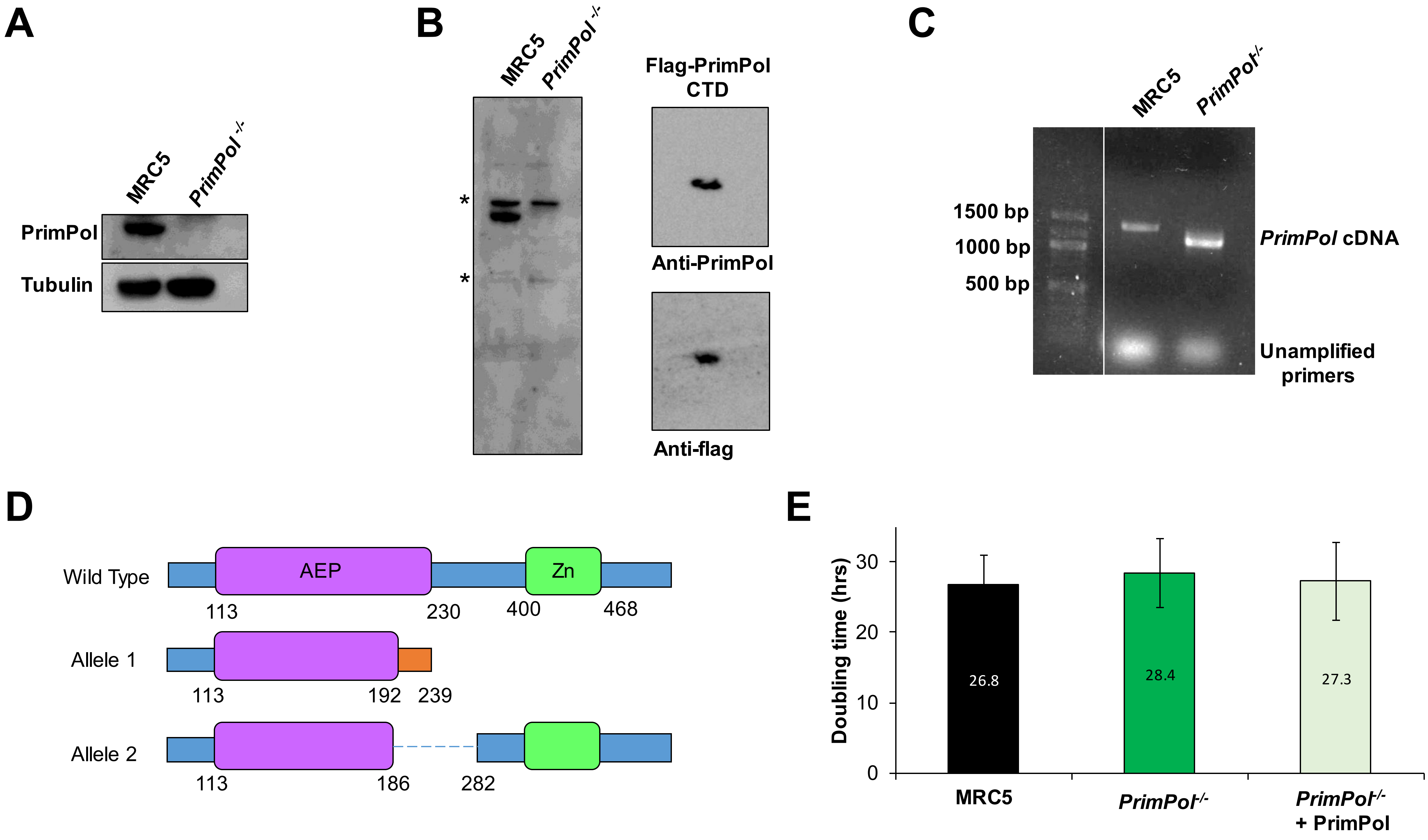

Supplementary Figure 2

Supplementary Figure 2. Generation of a PrimPol human knockout cell line.

Generation of MRC5 *PrimPol*$^{-/-}$ cells was confirmed using western blotting of whole cell lysate with a PrimPol specific antibody (**A**) against a tubulin control. (**B**) shows the extended western along with a western showing identification of a C-terminal truncated form of PrimPol, * denotes none specific bands. (**C**) rtPCR analysis identified only truncated forms of *PrimPol* mRNA in *PrimPol*$^{-/-}$ cells. Genomic DNA changes were identified by sequencing and these are depicted in cartoon form, amino acid numbers shown below (**D**). Doubling time was analysed by counting cell numbers over increasing time in both WT and *PrimPol*$^{-/-}$ cells or those complimented with GFP PrimPol, n≥3 with standard deviation shown as error bars (**E**).

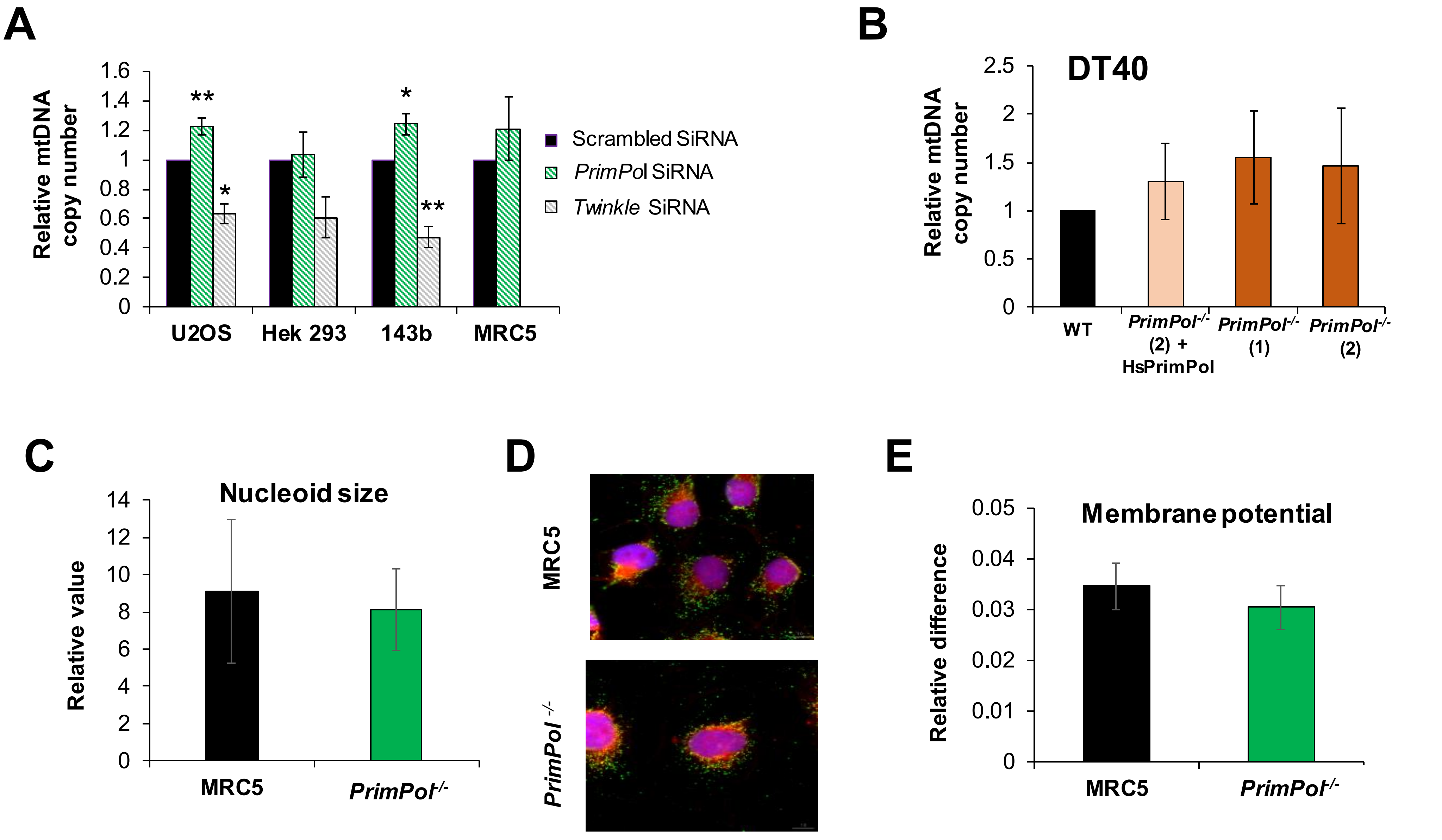

Supplementary Figure 3

Supplementary Figure 3. mtDNA copy number is increased in cells lacking PrimPol along with other replication changes.

Different cell lines were treated with siRNA targeted to *PrimPo*l or *Twinkle* or scrambled siRNA and mtDNA copy number was analysed by QPCR in comparison to the scrambled control (**A**), data is the mean of n≥3 (MRC5 n=2) with standard deviation represented by error bars. mtDNA copy number was also compared in WT DT40 chicken cells, two *PrimPol*$^{-/-}$ DT40 clones or those complimented with HsPrimPol (described in (10)) (**B**). *PrimPol*$^{-/-}$ and MRC5 control cells were used to analyse mtDNA organisation and functionality, nucleoid size was measured using Image J cells stained with Mitotracker and anti-DNA (**C**), representative images shown in (**D**), Mitotracker stained mitochondria, red, mtDNA nucleoids stained with antiDNA antibody green and nucleus DAPI, shown in blue. Mitochondrial membrane potential was measured using Mitotracker by comparing staining with a mass specific, membrane potential independent dye and a membrane dependent dye using flow cytometry (**E**).

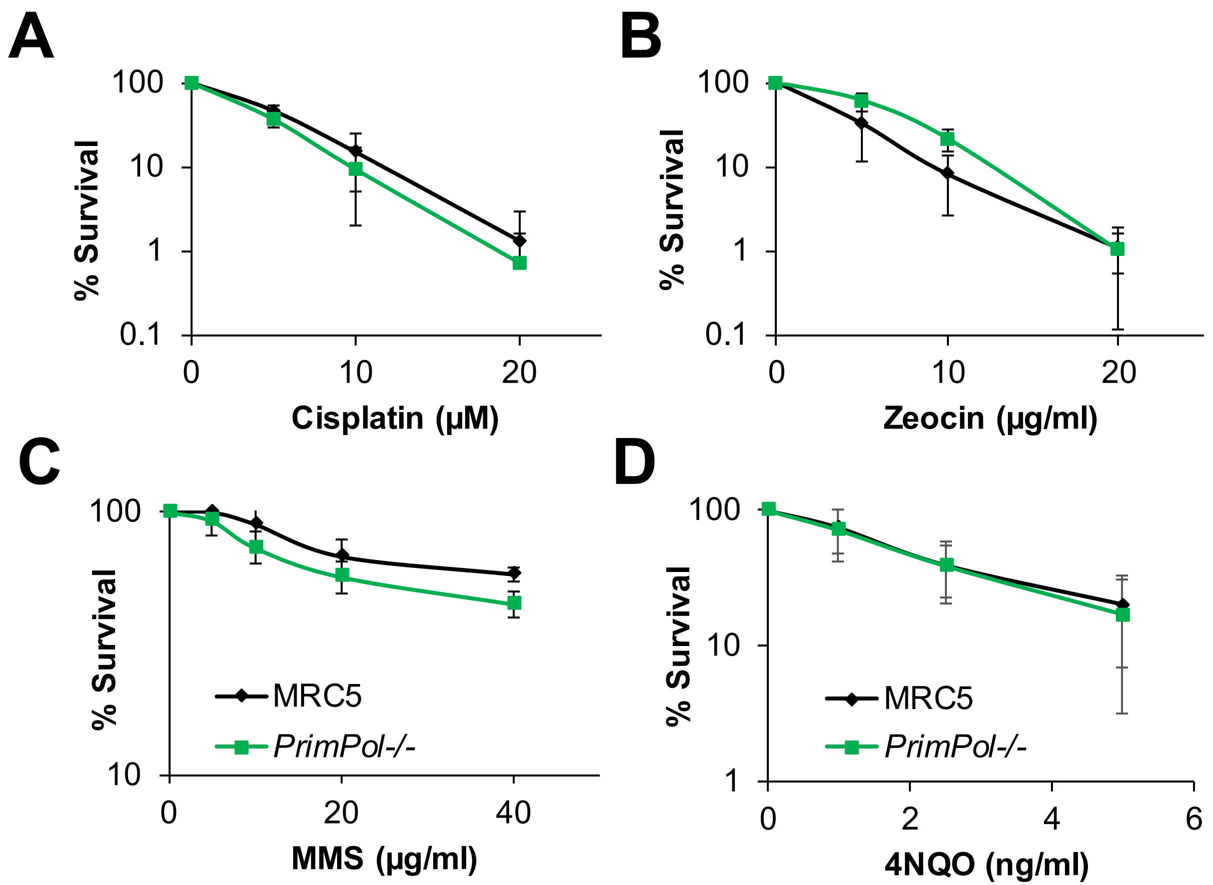

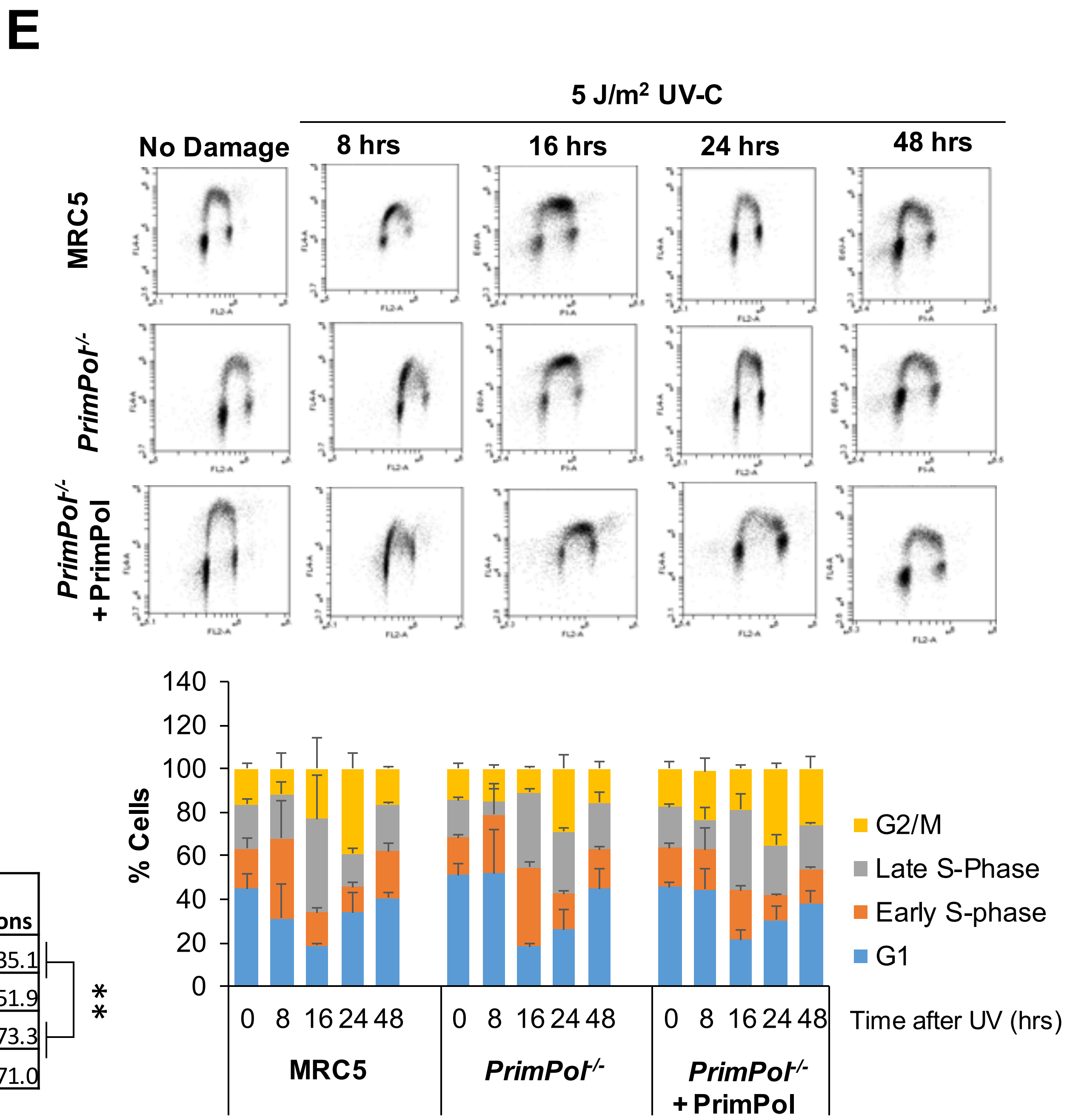

F

Cell line	Damage	Clones sequenced	% WT	% Point mutations	% Exon loss	% Insertions	% Transitions	% Transversions
MRC5	4NQO	48	6.6	59.0	24.6	9.8	64.9	35.1
MRC5	UV-C	44	21.7	63.0	15.2	0.0	48.1	51.9
PrimPol-/-	4NQO	40	13.3	35.6	42.2	8.9	26.7	73.3
PrimPol-/-	UV-C	48	18.4	59.2	22.4	0.0	29.0	71.0

**

Supplementary Figure 4

Supplementary Figure 4. *PrimPol$^{-/-}$* cells are not sensitive to DNA damaging agents but have an increased recovery time. *PrimPol$^{-/-}$* cells showed no significant increase in sensitivity when treated with a range of DNA damaging agents, Cisplatin, Zeocin, methylmethanosulphate (MMS) and 4NQO. (**A, B, C, D**), charts represent n=3 or more independent experiments with standard deviation shown as error bars. (**E**) Representative images of flow cytometry of EdU and propidium iodide stained cells at increasing time points after 5 J/m^2 UV-C damage quantified in Figure 2E. A full quantification of the cell cycle profiles is shown in the lower panel, n= 3 with standard deviation shown as error bars. (**F**) shows details of the *HPRT* clones screened for mutations. P= 0.0086 using a two-way ANOVA for changes in transitions and transversions between *PrimPol$^{-/-}$* and WT MRC5 cells after damage.

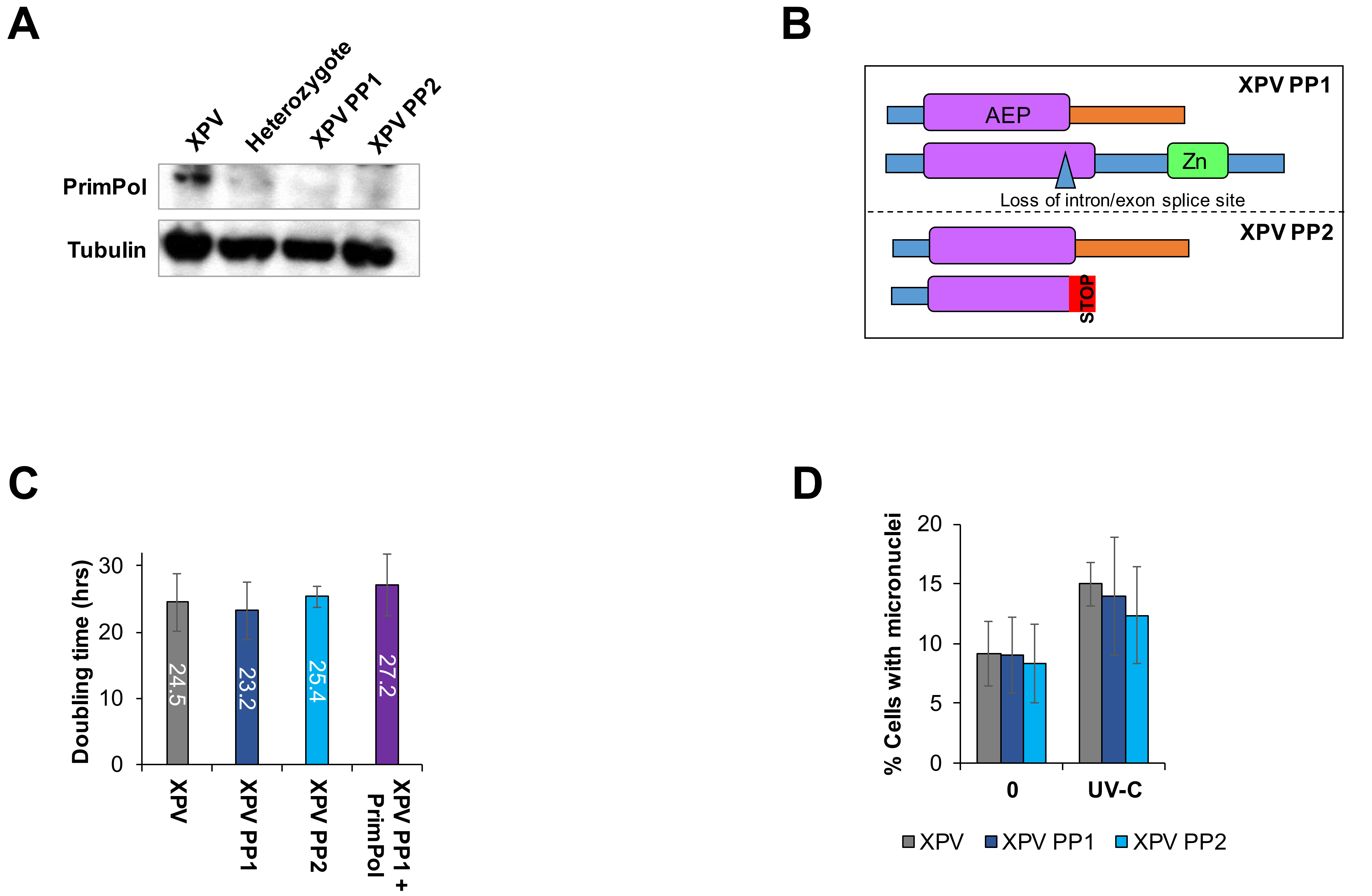

Supplementary Figure 5

Supplementary Figure 5. Generation of *PrimPol$^{-/-}$* cells in the *Pol* $\eta^{-/-}$ XP30RO cell line. (**A**) Two XP30RO clones lacking PrimPol protein expression were identified by western blot of whole cell lysate with a PrimPol specific antibody along with tubulin control, XP PP1 and XP PP2. (**B**) shows the genomic changes observed in the two *PrimPol$^{-/-}$* clones in the XP30RO causing the loss of the protein. (**C**) Growth rates were analysed in WT XPV cells along with the *PrimPol$^{-/-}$* clones and those complimented with GFP PrimPol by counting on a hemacytometer. (**D**) Cells were stained with DAPI 72 hrs after 0 or 2J/m^2 UV-C and cells with 1 or more micronuclei were counted as a percentage of the whole population.

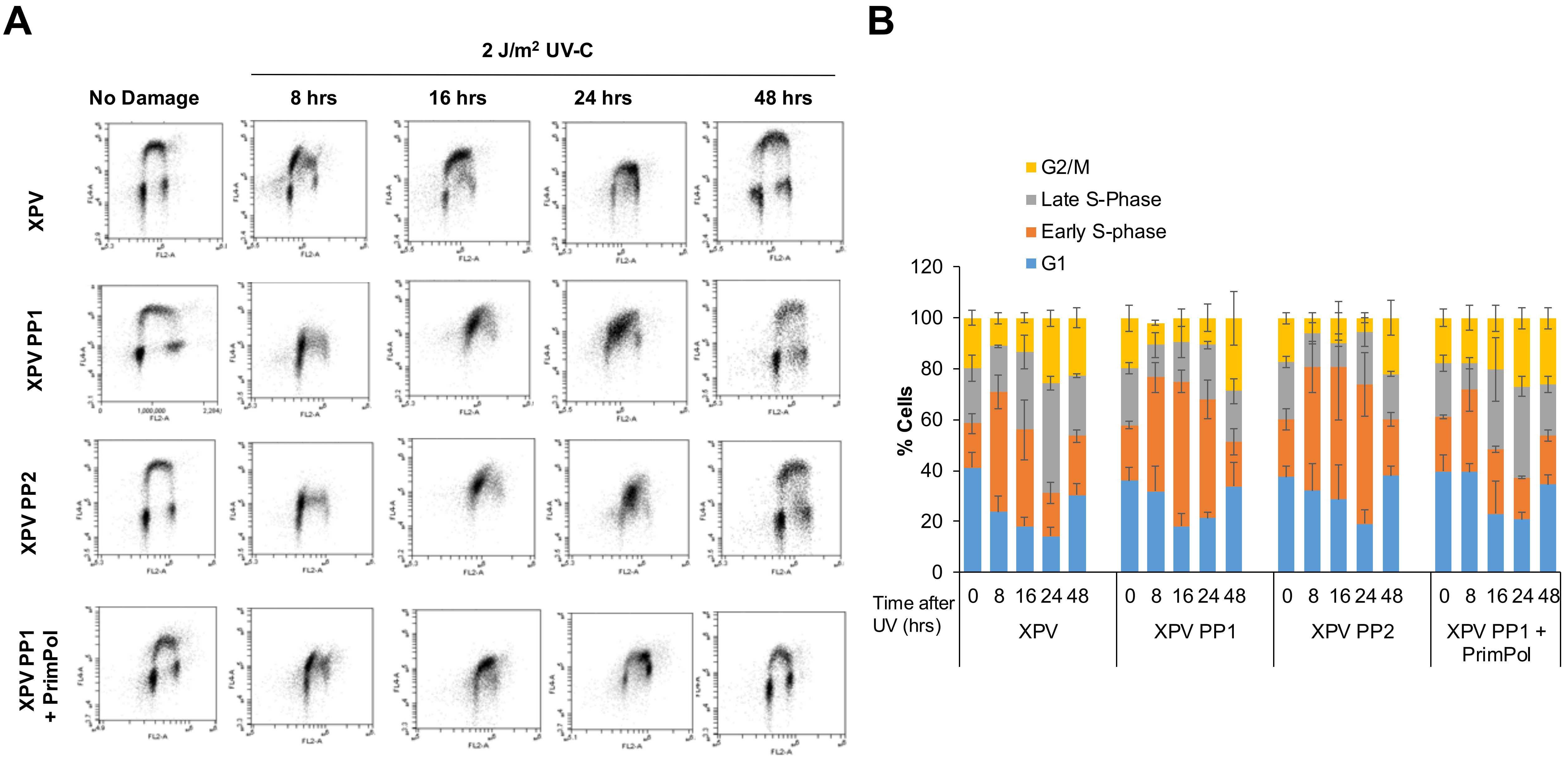

Supplementary Figure 6

Supplementary Figure 6. XP PP cells have delayed recovery after UV-C damage. (**A**) shows representative images of flow cytometry analysis of cells stained with EdU at increasing time points after 2 J/m^2 UV-C, quantified Fig. 4E. Full quantification of the cell cycle profiles shown in (**B**) $n \geq 3$ with standard deviation shown as error bars.
